# Association between body mass index and return to work following primary knee arthroplasty: a population-based cohort study on 6,128 patients from Danish national registers

**DOI:** 10.2340/17453674.2025.44253

**Published:** 2025-07-13

**Authors:** Julie B PAJANIAYE, Peter ALSING, Martin G STISEN, Erzsébet HORVÁTH-PUHÓ, Maaike G J GADEMAN, Alma B PEDERSEN, Inger MECHLENBURG

**Affiliations:** 1 Department of Clinical Epidemiology, Aarhus University Hospital, Denmark; 2 Department of Orthopaedic Surgery, Aarhus University Hospital, Denmark; 3 Department of Dentistry and Oral Health, Aarhus University, Denmark; 4 Department of Clinical Medicine, Aarhus University, Aarhus, Denmark; 5 Center for Population Medicine, Aarhus University and Aarhus University Hospital, Denmark; 6 Department of Orthopaedics, Leiden University Medical Centre, University of Leiden, Leiden, the Netherlands; 7 Department of Clinical Epidemiology, Leiden University Medical Centre, University of Leiden, Leiden, the Netherlands; 8 Research Center for Activity and Prevention, VIA University College, Aarhus, Denmark

## Abstract

**Background and purpose:**

With more knee arthroplasties (KAs) performed in working-age patients, interest in return to work (RTW) increases. We aimed to investigate the association between body mass index (BMI) and RTW after primary KA and whether the association varies by sex, age, comorbidity, and socioeconomic position.

**Methods:**

From Danish national registries, we included 6,128 patients aged 18 to 60 years undergoing KA from 2008–2018. Exposure was BMI in categories < 25.0, 25.0–29.9, 30.0–34.9, 35.0–39.9, and ≥ 40.0. Outcome was RTW after KA. We estimated cumulative incidence proportions (CIP) of RTW. Cox regression was used to calculate hazard ratios (HRs) with 95% confidence intervals (CI).

**Results:**

Median time to RTW was 70 days. Overall CIP for RTW was 63% (CI 62–65) at 3 months. With BMI < 25 as reference, CIP was 65% (n = 1,401) for BMI 25.0–29.9, 64% (n = 1,130) for BMI 30.0–34.9, 60% (n = 528) for BMI 35.0–39.9, and 60% (n = 260) for BMI ≥ 40.0, corresponding to an adjusted HR of 1.06 (CI 0.98–1.15), 1.02 (CI 0.94–1.11), 0.97 (CI 0.88–1.06), and 0.96 (CI 0.85–1.08). Men with BMI 35.0–39.9 and ≥ 40 had an adjusted HR of 0.89 (CI 0.76–1.05) and 0.87 (CI 0.70–1.10). None of the associations were statistically significant. Age, comorbidity, and socioeconomic position did not modify the association between BMI and RTW.

**Conclusion:**

More than 60% of patients RTW within 3 months but we found no association between BMI and RTW after primary KA.

With a growing number of knee arthroplasties (KAs) being performed on working-age individuals [1,2], there is increasing interest in their return to work (RTW) following surgery [3-7]. As a primary objective of KA is to restore patients’ functional abilities, the RTW after KA is crucial for patients, healthcare systems, and society [7]. However, a recent systematic review found considerable variation in the reported rates and average time to RTW across countries [7]. These inconsistencies could partly be explained by international differences in patient guidance and healthcare systems [5] but may also be due to small sample sizes and differences in definitions of RTW.

In Denmark, 20% of KAs are performed on patients under the age of 60 [8]. While the quality of surgical procedures and length of stay are well documented in high-quality registries [8-10], there is limited information on the expected time and rate of RTW after KA [5]. Data from our national registries could help fill this knowledge gap and address the limitations of previous studies.

Obesity is a modifiable risk factor for the development of knee osteoarthritis [2], poor function, low quality of life, and complications following KA [11]. With more younger patients undergoing KA [1] and rising obesity rates [12], it is likely that obesity is associated with delayed time and reduced rate of RTW. Since obesity is closely related to social inequality, comorbidity, age, and sex, these factors may be associated with RTW [2,7].

We aimed to examine the association between body mass index (BMI) and RTW in patients aged 18–59 years over a 2-year follow-up period after KA. Additionally, we explored whether this association varied by socioeconomic position, comorbidity, age, and sex.

## Methods

### Study design

We conducted a population-based cohort study with prospectively collected data, reported in adherence to the STrengthening the Reporting of OBservational Studies in Epidemiology (STROBE) guideline.

### Data sources

We retrieved data from the Danish Knee Arthroplasty Register (DKR) [9], the Danish Register for Evaluation of Marginalization (DREAM) [13], the Danish Civil Registration System, the Danish National Patient Register, and social registries of Statistics Denmark [14]. These registries were linked using the unique civil personal registration number assigned to all Danish residents.

### Study population

The DKR provides data on KAs from all Danish public and private hospitals covering > 90% of procedures with > 95% completeness, including information on indication for KA and preoperative objective recording of height and weight [8,9]. We used the DKR to identify patients with knee osteoarthritis undergoing KA between 2008 and 2018. We considered total knee arthroplasty and unicompartmental knee arthroplasty equally relevant to examine for the study, as they have equal length of stay and recovery patterns [10,15] and therefore analyzed these implant types under the term KA. We included the first primary KA of patients aged 18 to 59. The date of the primary KA was defined as the index date. Subsequent contralateral primary KA or revision in the same patient was not analyzed. Exclusion criteria were < 18 and ≥ 60 years of age at the index date, as early retirement schemes were available for those 60 years and older. Other exclusion criteria included prior early retirement, non-osteoarthritis KA indications, primary KAs misclassified as revision surgeries in the DKR, missing BMI data, and negative family income.

### Body mass index

BMI was measured at the index date. To account for the effect of different levels of BMI, we categorized BMI according to the ICD-11 WHO classification [16]. Due to less than 20 underweight patients, the underweight and normal weight categories were combined. This resulted in 5 BMI categories: normal weight (BMI < 25.0), pre-obesity (BMI 25.0–29.9), obesity class I (BMI 30.0–34.9), obesity class II (BMI 35.0–39.9), and obesity class III (BMI ≥ 40).

### Return to work

We used the DREAM register to assess patients’ labor market status 6 months and 4 weeks prior to the index date, as well as their recovery from sick leave to RTW after KA. We retrieved weekly data from the DREAM register for all KA patients in the study population. The DREAM register contains weekly data on individual-level public transfers of social benefits, sick leave or unemployment benefits, pensions, and information on labor market status, death, or emigration for all Danish citizens, and has a high degree of accuracy and completeness [13]. In line with existing literature [17,18], RTW was defined as the point at which a patient either did not receive any transfer payments for 4 consecutive weeks, indicating employment, or received unemployment benefits for 4 consecutive weeks, indicating availability to work, whichever occurred first. Details on categorization of the DREAM codes can be found in Table S1 (see Supplementary data). Follow-up was counted in days from the index date for a period of 24 months (730 days) until RTW, censoring, or a competing event, whichever occurred first. Competing events included voluntary or early retirement and death. Failure to achieve RTW was defined by continuous DREAM sick leave codes. By law, the first weeks of a sick leave period are covered by the employer, and this period is not recorded in DREAM. The duration of the employer-paid sick leave period varied from 2 to 4 weeks in the study period (Table S1, see Supplementary data). An absence of DREAM codes for up to 4 weeks after the index date may indicate no sick leave or an employer-paid sick leave period. We assigned an RTW time of 14 days to patients with no DREAM codes for 4 weeks post-index date.

### Covariates

Sex and age of the cohort members at the index date were retrieved from the Danish Civil Registration System. Age was categorized into groups of 5-year intervals: < 45, 45–49, 50–54, and 55–59 years. Data on the type of household recorded in the Danish Civil Registration System as of 1 October of the year prior to the index date was used to dichotomize patients as living alone or cohabiting.

The Danish National Patient Register provides comprehensive information from Danish hospitals on a wide range of data, comprising discharge diagnoses. We retrieved information on comorbidities using discharge diagnosis codes from the Danish National Patient Register at any time before the index date. Disease categories were assigned weights based on their severity to calculate Charlson Comorbidity Index (CCI) scores, which range from 0 to 12. CCI scores were grouped into low (score of 0), medium (score of 1–2), or high (score of ≥ 3).

Data on annual household income, type of employment, and highest education attained were retrieved from Statistics Denmark. Family income is registered annually in the registries of Statistics Denmark at the end of each calendar year as the sum of the household’s disposable income, including children aged < 25 living with the family, adjusted for differences between renters and homeowners. We used the average annual family income of the 5 calendar years preceding the index date, split according to tertiles of the income distribution. Type of employment was categorized as director/chief executive, employer/self-employed, skilled worker, unskilled worker, and other/unknown, based on the status registered in November of the year before the index date. Highest attained education was categorized as low (primary or lower secondary education), medium (upper secondary or academic profession degree), or high (university education at bachelor’s level or above) at the end of the year before the index date.

We merged data on cohabitation status, education, and family income to create a composite measure of socioeconomic position prior to the index date [19]. We assigned scores of 1–3 (low to high) to education level and family income tertile, and scores of 1 (living alone) or 2 (cohabiting) to cohabitation status. The sum of scores was used to categorize socioeconomic position as low (scores 3–4), medium (scores 5–6), or high (scores 7–8). If values were missing for any variable, socioeconomic position was not computed.

### Statistics

Prevalences of categorical and dichotomous variables were presented as numbers and percentages. Non-normally distributed continuous data were presented as median and interquartile range (IQR), from the 25th to 75th percentile. The median time to RTW and IQR was presented as time in days since the index date for the total population and for BMI groups. Confidence levels were set at 95%, and the corresponding confidence intervals were presented as CI.

The Aalen–Johansen estimator was used to compute the cumulative incidence proportions (CIP) of RTW at 1, 3, 6, 12, and 24 months with death and early retirement as competing events for the total study population and for BMI groups. Censoring occurred in the case of emigration.

We used a Cox proportional hazards analysis to estimate the hazard ratios (HRs) for RTW with 95% CIs for the entire study population and by BMI groups during a 24-month follow-up period to estimate the association between BMI and RTW at selected time points deemed relevant for patients and society. We assessed the fulfillment of the proportional hazards assumption using visual inspections of log-minus-log plots. We estimated crude and adjusted HRs (aHRs) using the pre-specified selected confounders presented in the directed acyclic graph (Figure S1, see Supplementary data). Adjusted analyses comprised only patients with no missing data on covariates. We performed stratified analyses by age group, sex, socioeconomic position, and comorbidity group to account for underlying mechanisms. We performed sensitivity analyses to explore the robustness of our model. First, we used the adjusted Cox proportional hazards model to calculate a series of aHRs with increasing follow-up time to explore whether these would be more informative than the average aHRs with 2 years of follow-up. Second, we used BMI as a continuous variable in an adjusted Cox proportional hazards regression analysis. Data management and statistical analysis were performed using Stata 18 (StataCorp 2023; StataCorp LLC, College Station, TX, USA).

### Ethics, registration, data sharing plan, funding, use of AI tools, and disclosures

Approval from an ethics committee is not required for register-based studies in Denmark. The study was reported to the Danish Data Protection Agency through registration at Aarhus University (record number: AU-2016-051-000001, sequential number 880). Patients or the public were not involved in the planning of the study. Data from the project stems from Danish registries containing personal information and cannot be shared. The original data is only handled on secure servers of Statistics Denmark, and solely aggregated results can be disseminated. The study was funded by the Department of Clinical Epidemiology, Aarhus University, Denmark. AI tools were not used. The authors have no conflicts of interest to declare. Complete disclosure of interest forms according to ICMJE are available on the article page, doi: 10.2340/17453674.2025.44253

## Results

### Study population and characteristics

**Figure 1:**
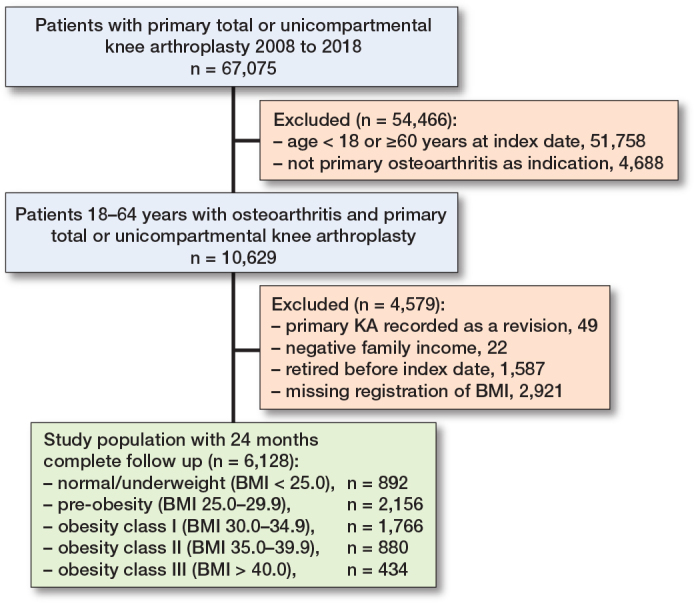
Participant flowchart. KA: knee arthroplasty. Index date: date of knee arthroplasty. BMI: body mass index.

From a total of 67,075 patients undergoing KA between 2008 and 2018, 6,128 were included in the final cohort (Figure 1). No patients had missing data in the follow-up period. Most patients (77%) were excluded due to age restrictions, while 4.4% were excluded due to missing BMI data (Figure 1). BMI recording in the DKR was not mandatory until mid-2011, resulting in a high degree of missing data from 2008–2010 (Table S2, see Supplementary data). Patients excluded due to missing BMI had baseline characteristics and outcome measures similar to those included in the final cohort (Table S2, see Supplementary data). In the final cohort, the proportion of women was 59% and the median age was 55.1 years (IQR 51.5–57.8) (Table 1). Patient characteristics between BMI groups did not differ substantially; however, the proportion of patients diagnosed with diabetes increased with increasing BMI, as did the proportion of patients in lower socioeconomic positions (Table 1). 6 months before KA, the overall proportion of patients on sick leave was 9.4%, and the proportions did not differ substantially between BMI groups. At 1 month before the index date, the overall proportion was rising to 17.4% and proportions were somewhat increasing with increasing BMI (Table S3, see Supplementary data). Employment status was used for descriptive purposes only, as 22% of the population was classified as other/unknown (Table S3, see Supplementary data).

**Table 1:** Baseline characteristics of included patients. Values are counts (%) unless otherwise specified

Item	All	Normal weight	Pre-obesity	Obesityclassl	Obesityclassll	Obesityclasslll
Number of patients	6,128	892(15)	2,156 (35)	1,766 (29)	880 (14)	434 (7)
Sex						
Male	2,492 (41)	343 (39)	1,041 (48)	718 (41)	271 (31)	119 (27)
Female	3,636 (59)	549 (62)	1,115 (52)	1,048 (59)	609 (69)	315 (73)
Median age at KA (IQR)	55.1 (51.5-57.8)	55.3 (52.1-57.8)	55.3 (51.7-57.8)	54.9 (51.2-57.8)	54.9 (51.3-57.7)	54.8 (50.9-57.6)
Age group at KA, years						
<45	273 (4.5)	42 (4.7)	93 (4.3)	81 (4.6)	36 (4.1)	21 (4.8)
45-49	804 (13)	108(12)	262 (12)	247 (14)	117 (13)	70 (16)
50-54	1,947 (32)	276 (31)	668 (31)	574 (33)	296 (34)	133 (31)
55-59	3,104 (51)	466 (52)	1,133 (53)	864 (49)	431 (49)	210 (48)
Median weight, kg (IQR)	90 (80-103)	70(64-77)	84 (76-90)	95 (88-104)	108 (100-117)	123 (114-135)
Median BMI (IQR)	30.0 (26.6-34.2)	23.7 (22.4-24.4)	27.7 (26.4-28.8)	32.2 (31.1-33.5)	36.9 (35.9-38.3)	42.9 (41.0-45.1)
Type of implant						
TKA	4,375 (71)	591 (66)	1,520 (71)	1,229 (70)	679 (77)	356 (82)
UKA	1,404 (23)	249 (28)	516 (24)	426 (24)	156 (18)	57 (13)
Other/unknown	349 (5.7)	52 (5.8)	120 (5.6)	111 (6.3)	45 (5.1)	21 (4.8)
Side of operation						
Right	3,191 (52)	466 (52)	1,123 (52)	958 (54)	434 (49)	210 (48)
Left	2,937 (48)	426 (48)	1,033 (48)	808 (46)	446 (51)	224 (52)
Functional status						
Unilateral impairment	801 (13)	149(17)	295 (14)	226 (13)	90 (10)	41 (9.5)
Contralateral impairment	313 (5.1)	45 (5.0)	92 (4.3)	96 (5.4)	58 (6.6)	22 (5.1)
Contralateral prosthesis	121 (2.0)	17(1.9)	26 (1.2)	50 (2.8)	15(1.7)	13 (3.0)
Otherfunctional impairment	96 (1.6)	10(1.1)	34 (1.6)	26 (1.5)	12 (1.4)	14 (3.2)
Missing	4,797 (78)	671 (75)	1,709 (79)	1,368 (78)	705 (79)	344 (79)
Region						
Capital	1,880 (31)	285 (32)	727 (34)	496 (28)	237 (27)	135 (31)
Zealand	1,353 (22)	170(19)	475 (22)	410 (23)	201 (23)	97 (22)
Southern	1,316 (22)	187 (21)	441 (21)	371 (21)	213 (24)	104 (24)
Central	1,036 (17)	178 (20)	352 (16)	318 (18)	147 (17)	41 (9.5)
Northern	538 (8.8)	72 (8.1)	160 (7.4)	169 (9.6)	81 (9.2)	56 (13)
Charlson Comorbidity Index						
Low	5,267 (86)	758 (85)	1,866 (87)	1,527 (87)	750 (85)	366 (84)
Medium	783 (13)	121 (14)	271 (13)	216 (12)	115 (13)	60 (14)
High	78 (1.3)	13(1.5)	19 (0.9)	23 (1.3)	15(1.7)	8(1.8)
Most frequent comorbidities						
Diabetes	604 (9.9)	42 (4.7)	116 (5.4)	213 (12)	139 (16)	94 (22)
Cancer (any malignancy)	289 (4.7)	47 (5.3)	104 (4.8)	80 (4.5)	35 (4.0)	23 (5.3)
Chronic obstructive pulmonary disease	137 (2.2)	16(1.8)	32 (1.5)	52 (2.9)	26 (3.0)	11 (2.5)
Cohabitation status						
Living alone	1,277 (21)	199 (22)	450 (21)	339 (19)	189 (22)	100 (23)
Cohabiting	4,851 (79)	693 (78)	1,706 (79)	1,427 (81)	691 (79)	334 (77)
Median annual family income in x10^3^€(IQR)	86 (60-109)	88(60-111)	90 (64-112)	86 (59-110)	82 (56-104)	74 (52-94)
Annual family income groups, tertiles						
High	541 (8.8)	85 (9.6)	167 (7.8)	144 (8.2)	95 (11)	50 (12)
Medium	1,390 (23)	196 (22)	441 (21)	421 (24)	197 (23)	135 (31)
Low	4,168 (68)	607 (68)	1,540 (72)	1,191 (68)	585 (67)	245 (57)
Missing	29 (0.5)	-	-	-	-	-
Educational level						
Low	1,601 (27)	204 (23)	533 (25)	449 (26)	283 (33)	132 (32)
Medium	3,294 (55)	459 (53)	1,215 (57)	958 (56)	449 (52)	213 (51)
High	1,089 (18)	209 (24)	374 (18)	310 (18)	127 (15)	69 (17)
Missing	144 (2.3)	-	-	-	-	-
Socioeconomic position						
Low	534 (8.7)	84 (9.4)	173 (8.0)	135 (7.6)	96 (11)	46 (11)
Medium	2,351 (38)	302 (34)	799 (37)	685 (39)	366 (42)	199 (46)
High	3,087 (50)	486 (55)	1,147 (53)	890 (50)	396 (45)	168 (39)
Not computed	156 (2.5)	20 (2.2)	37 (1.7)	56 (3.2)	22 (2.5)	21 (4.8)

BMI: body mass index. KA: knee arthroplasty. TKA: total knee arthroplasty, UKA: unicompartmental knee arthroplasty. IQR: interquartile range, 25th to 75th percentile. Categorization of BMI according to WHO ICD-11 classification: Normal weight and underweight (BMI < 25.0), pre-obesity (BMI 25.0-29.9), obesity class I (BMI 30.0-34.9), obesity class II (BMI 35.0-39.9), and obesity class III (BMI ≥ 40). Count (%) for missing data on annual family income groups and educational level is shown only for the overall population due to low numbers in BMI subgroups. Annual income: average of family income of the 5 calendar years preceding the index date. Categories of socioeconomic position created from educational level, cohabitation status, and family income tertile by assigning scores of 1-3 (low to high) to education level and family income tertile, and scores of 1 (living alone) or 2 (cohabiting) to cohabitation status. The sum of scores was used to categorize socioeconomic position as low (scores 3-4), medium (scores 5-6), or high (scores 7-8). If values were missing for family income and/or educational level, socioeconomic position was not computed.

### Return to work

**Figure 2:**
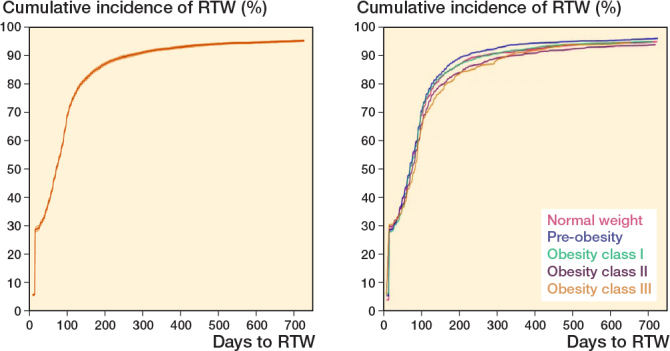
Cumulative incidence curves (%) of return to work (RTW) for the total population (left panel) and by body mass index (BMI) group (right panel) in days after total knee arthroplasty. For BMI classification, see Table 1.

The overall median time to RTW after KA was 70 days (IQR 7–111) and ranged from 69 days (IQR 7–107) for patients in the normal BMI group to 82 days (IQR 7–130) for patients in obesity class III (Table 2). At 1 month, the overall CIP of RTW was 31% (CI 30–32), which increased to 63% (CI 62–65) at 3 months after KA (Figure 2). At the end of the 24-month follow-up, 95% (CI 95–96) of the cohort had RTW (Table 3). There was no difference in RTW between BMI groups (Table 4).

**Table 2:** Median (IQR) days to return to work (RTW) after knee arthroplasty overall and by body mass index (BMI) groups

Population	Median RTW (IQR)
Overall	70 (7 to 111)
BMI group	
Normal weight	69 (7 to 107)
Pre-obesity	69 (7 to 108)
Obesity class 1	70 (7 to 110)
Obesity class II	76 (7 to 119)
Obesity class III	82 (7 to 130)

For BMI classification and IQR, see Table 1.

**Table 3:** Cumulative incidence proportions (CIP) (%) with 95% confidence intervals (CI) of return to work after knee arthroplasty at 1, 3, 6, 12, and 24 months for the total population and according to body mass index (BMI) groups. Values include number of outcome events in the cohort

			Time after knee arthroplasty							
Population	1 month		3 months		6 months		12 months		24 months	
	n	CIP % (Cl)	n	CIP % (Cl)	n	CIP % (Cl)	n	CIP % (Cl)	n	CIP % (Cl)
Overall 1	1,900	31 (30-32)	3,861	63 (62-65)	5,270	86 (85-87)	5,699	93 (92-93)	5,822	95 (95-96)
BMI group										
Normal weight	277	31 (28-34)	571	64 (61-67)	767	86 (83-88)	812	91 (90-93)	847	95 (94-96)
Pre-obesity	668	31 (29-33)	1,401	65 (63-67)	1,897	88 (87-89)	2,027	94 (93-95)	2,070	96 (96-97)
Obesity class I	530	30 (28-33)	1,130	64 (62-66)	1,519	86 (84-87)	1,625	92 (91-93)	1,678	95 (94-96)
Obesity class II	282	32 (29-35)	528	60 (57-63)	730	83 (81-86)	254	90 (88-92)	827	94 (92-95)
Obesity class III	139	32 (27-36)	260	60 (56-65)	356	82 (78-85)	395	91 (88-94)	412	95 (92-97)

For BMI classification, see Table 1.

**Table 4:** Crude and adjusted hazard ratios (HR) with 95% confidence interval (CI) for return to work within 24 months according to different body mass index (BMI) groups and stratified by sex. Values include number of subjects in the cohort

Population	n	Crude HR (Cl)	n	aHR ^a^ (CI)
Overall	6,128		5,972	
BMI group				
Normal weight	892	1	872	1
Pre-obesity	2,156	1.06 (0.98-1.15)	2,119	1.06 (0.98-1.15)
Obesity class 1	1,766	1.01 (0.92-1.09)	1,710	1.02 (0.94-1.11)
Obesity class II	880	0.94 (0.86-1.04)	858	0.97 (0.88-1.06)
Obesity class III	434	0.91 (0.81-1.02)	413	0.96 (0.85-1.08)
Males	2,492		2,423	
Normal weight	343	1	334	1
Pre-obesity	1,041	0.96 (0.85-1,09)	1,024	0.97 (0.86-1.11)
Obesity class 1	718	0.98 (0.86-1.12)	692	1.00 (0.88-1.14)
Obesity class II	271	0.87 (0.74-1.02)	263	0.89 (0.76-1.05)
Obesity class III	119	0.80 (0.64-0.87)	110	0.87 (0.70-1.10)
Females	3,636		3,549	
Normal weight	549	1	538	1
Pre-obesity	1,115	1.12 (1.01-1.24)	1,095	1.13 (1.02-1.26)
Obesity class 1	1,048	1.01 (0.91-1.13)	1,018	1.04 (0.93-1.15)
Obesity class II	609	0.99 (0.88-1.12)	595	1.01 (0.90-1.14)
Obesity class III	315	0.98 (0.85-1.12)	303	1.01 (0.87-1.16)

a aHR adjusted hazard ratio. Reference: normal weight group. Overall hazard ratios adjusted for sex, age, Charlson Comorbidity Index (CCI), and socioeconomic position. Stratified hazard ratios adjusted for age, Charlson Comorbidity Index, and socioeconomic position. Differences in number of patients from crude to adjusted HRs due to missing values on socioeconomic position. BMI: body mass index. For BMI classification, see Table 1.

When stratified by sex, the aHRs for women across BMI groups remained consistently similar to normal BMI, while the subgroup of men in obesity classes II and III had aHRs of 0.89 (CI 0.76–1.05) and 0.87 (CI 0.70–1.10) respectively (Table 4). Stratification by age groups, CCI, or socioeconomic position did not show any changes in aHRs compared with the overall analysis except for patients in obesity class III with a high CCI, a stratum containing < 10 patients (Table S4, see Supplementary data).

In a sensitivity analysis, we calculated the aHR for the association between BMI and RTW for several specific time periods after KA (6, 12, and 18 months), which did not show any changes in aHRs compared with the aHRs at 24 months’ follow-up (data not shown). Sensitivity analysis using BMI as a continuous measure did not change the overall results. A 10-unit increase in BMI was not associated with a decrease in the aHR of RTW (aHR 1.0, CI 0.9–1.0). A stratified analysis by sex showed that a 10-unit increase in BMI could decrease the aHR of RTW to 0.9 (CI 0.9–1.0) for men, while the aHR for women remained 1.0 (CI 0.9–1.0).

## Discussion

Our study is the first to report the time to and rate of RTW adjusted for sex, age, CCI, and socioeconomic position in a working-age nationwide population undergoing KA. We aimed to investigate the association between BMI and RTW after primary KA and whether the association varies by sex, age, comorbidity, and socioeconomic position. We found a slightly numerically longer time to RTW for obesity classes II and III (BMI ≥ 35) compared with all other BMI groups. Even though we did not find statistically significant associations between BMI and RTW after adjusting for potential confounders, we did observe a likely clinically relevant trend towards delayed RTW with increasing BMI groups. Our data suggests that men with the highest obesity class II or III have delayed RTW compared with men with normal BMI. The lower limit of the CIs does not exclude clinically important reduced rates of RTW of up to 25% or 30%, respectively, compared with men with a normal BMI.

The RTW proportions found in our study align with those reported in existing literature. A systematic review based on 14 small sample studies found RTW proportions ranging from 71% to 98% for the included studies, except for 1 study where the RTW proportion was 40% [7].

As recovery is expected for most patients within the first 3 months after KA [5], RTW proportions at 3 months are of particular interest for both patients and clinicians as an indicator of successful recovery, while the long-term proportions at 12 and 24 months provide information relevant to the continuous labor market attainment of the KA population.

The lack of a statistically significant overall association between BMI and RTW may be due to the selection of healthy patients with high BMI for KA. Patients with a high BMI, who are otherwise healthy and have stable employment, may be more likely to receive KA, and, in our cohort, the proportion of patients with a high CCI score was very low. The role of sex-specific obesity in RTW remains unclear, with limited studies yielding contradictory findings. One study found female sex and obesity to be negatively associated with RTW alongside knee-strenuous employment [20], while another study found no association [21]. Other studies suggest men have a higher probability of RTW, irrespective of obesity [22]. The overall role of sex in post-KA recovery is inconclusive [23], with several studies finding no sex-specific differences in RTW. Knee-strenuous jobs are negatively associated with RTW [11), and social and workplace support are important for facilitating RTW [24,25]. The contribution of society-level factors in influencing RTW, such as labor market organization, access to post-KA rehabilitation, paid sick leave schemes, and social security programs, should also be considered as possible structural factors that may shape and affect patients’ behavior post-KA [5,23,25], potentially even to an extent that may outweigh individual-level exposures. The large discrepancies in RTW proportions among different studies may not only result from small sample sizes and low methodological quality but also reflect such structural differences between countries.

Our finding that men in the highest BMI class experience delayed RTW may be explained by their higher likelihood of holding knee-strenuous jobs with limited rehabilitation options requiring full recovery before RTW. This negative association is particularly evident for men in higher BMI classes, who might have slower recovery patterns as they are employed in roles that do not permit a gradual return through work from home, lighter duties, or office-based tasks [24]. This underscores the significant impact of occupational class, especially knee-strenuous and physically demanding jobs, on RTW timing and the potential benefits of more comprehensive rehabilitation efforts than existing self-led rehabilitation exercise programs. Such efforts should be aimed at tailoring individualized postoperative recovery programs including intensive physiotherapy-led exercise and occupational counseling to facilitate RTW. Men with high BMI could also benefit from tailored preoperative assessments including identification of pre-existing comorbidities also known to negatively impact surgery outcome, assessment of the patient’s ability to perform daily activities, and their overall fitness level to be able to perform rehabilitation post-surgery, and discussion of the risks and benefits of surgery and how pre-surgery lifestyle modifications can improve their post-surgery outcome.

### Strengths

We used nationwide register data, with a high degree of completeness [9] and low risk of information bias, allowing us to include a large cohort with complete follow-up. This cohort, including both total and unicompartmental knee arthroplasties, reflects the current trend in arthroplasty practice where BMI distribution, length of stay, and recovery patterns are clinically similar across these treatment modalities [10,15]. This cohort is potentially more representative of the population compared with previous questionnaire-based RTW studies, where selection bias may exclude patients in less favorable positions, including those with severe obesity. Furthermore, the inclusion and exclusion criteria helped avoid the introduction of selection bias. As patients in higher obesity classes may be more likely to be unemployed prior to their KA, we avoided the exclusion of patients in unemployment, social, or sick leave benefit categories. We only excluded patients on early retirement schemes and included both self-supporting patients and those receiving unemployment, social, or sick leave benefits before the index date, as these patients would hold the potential to become self-supporting post-KA. Lastly, we were able to apply a consistent definition of RTW based on valid and reliable data sources [13].

### Limitations

We had to exclude patients due to missing BMI data. However, the missingness seems to be a calendar year effect rather than a result of selection bias. The proportion of patients on sick leave at 1 month prior to the index date was slightly higher for higher BMI groups, which may have led to residual confounding of our estimates.

Furthermore, we were unable to distinguish between the proportions of full and partial RTW, or the number of hours worked for partial RTW. Finally, unmeasured confounding like specific knee-strenuous occupations, workplace and social support, and other individual incentives to return to work, which have been identified by other studies as significant factors for RTW after KA [7,23-25] are not captured in Danish registers.

### Conclusion

In this cohort, more than 60% of patients returned to work within 3 months after KA. Overall, BMI was not associated with delayed time to and reduced rate of RTW.

### Supplementary data

Tables S1–S3 and Figure S1 are available as supplementary data on the article page, doi: 10.2340/17453674.2025.44253

## Supplementary Material



## Notes

JBP drafted the manuscript. JBP, PA, MGS, EHP, MGJG, ABP and IM conceived and designed the study, interpreted the results, and revised the manuscript.

Handling co-editors: Marianne Westberg and Robin Christensen

*Acta* thanks Mona Badawy and Petri Virolainen for help with peer review of this manuscript.
