# Supplementary material for: Association between body mass index and return to work following primary knee arthroplasty: a population-based cohort study on 6,128 patients from Danish national registers
Source: Acta Orthop. 2025 Jul 13;96:529–36. doi: 10.2340/17453674.2025.44253 (PMC12257360; doi:10.2340/17453674.2025.44253)
Supplement: Supplementary file 1 [file ActaO-96-44253-s1.pdf]

Supplementary data

Table S1. Supplementary information on the Danish Register for Evaluation of Marginalization (DREAM) data sources and coding

Present and historic DREAM codes 2008–2020	Type of transfer payment	Category
784 785<br>810 811 812 813 814 815 816 817 818<br>870 871 872 873 874 875 876 877 878<br>890 891 892 893 894 895 896 897 898	Sick leave benefits, workability clarification, vocational rehabilitation program for long-term sick leave	No RTW
“no entry”<br>122 123<br>899	Self-supporting, vacation payment from employment, partial self-supporting/partial sick-leave	RTW
521 651 652 661 662 794	State Education Fund grants, Trainee (adult)	RTW
881	Maternity leave	RTW
412 413 795	Leave-of-absence schemes due to sick child, extended maternity leave, or education	RTW
750 751 752 753 754 755 756 757 758<br>760 761 762 763 764 765 766 767 768 769<br>791 792	Employment or training in rehabilitation scheme	RTW
771 772 773 774 779 781 782 796	Flexible job for people with reduced workability	RTW
111 112 113 114 115 121 124 125 126<br>211 212 213 214 215 216 217 218 219 211<br>222 224 225 231 232 297 298 299<br>511 522 541<br>740 743 744 745 746 747 748 759	Unemployment benefits, all types, not health related	RTW
130 131 132 133 134 135 136 137 138 139<br>140 141 142 143 144 145 146 147 148 149<br>151 152 153 160 163 164 165 166 167 168<br>169<br>700 703 704 705 706 707 708 709 710 711<br>712 713 714 715 716 717 718 719 720 721<br>722 723 724 725 726 727 728 729 730 731<br>732 733 734 735 736 737 738 739 741 742	Social assistance, not health related	RTW
999	Death	Competing event
611 621 622	Voluntary early retirement, not health related	Competing event
783 793 797	Disability pension (health related)	Competing event
996 998	Retirement age, State Pension	Competing event
997	Not resident in Denmark	Censoring

RTW: return to work. DREAM codes are weekly codes on public transfers of social benefits, sick leave or unemployment benefits, pensions, as well as information on labor market status, death, or emigration for all Danish citizens used for categorization of the outcome. “No entry” indicates self-support. Employer-paid sick leave is not registered with codes in the DREAM database and is also consistent with “no entry.” The duration of employer paid-sick leave was 14 days until June 1, 2008; the duration was 21 days from June 1, 2008 until January 1, 2012; the duration was 30 days from January 1, 2012 and onwards.

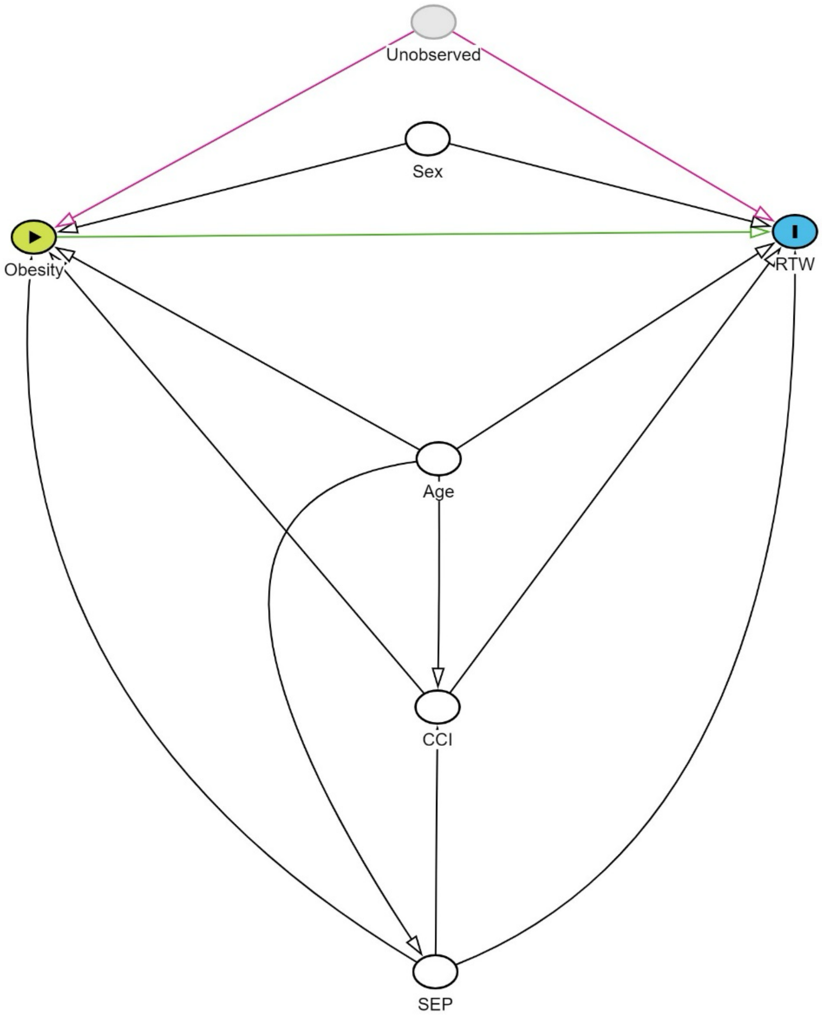

Figure S1. Directed acyclic graph of the model used for analysis. SEP: socioeconomic position. CCI: Charlson Comorbidity Index. RTW: return to work. Graph created using DAGITTY.NET (Johannes Textor, Benito van der Zander, Mark K. Gilthorpe, Maciej Liskiewicz, George T.H. Ellison. Robust causal inference using directed acyclic graphs: the R package "dagitty". Int J Epidemiol 2016; 45(6): 1887-94).

Table S2. Selected baseline characteristics and outcome measures of patients with and without body mass index recordings. Values are counts (%) unless otherwise specified

Item	Included patients with BMI data	Excluded patients without BMI data
Number of patients	6,128	2,921
Sex		
Male	2,492 (41)	1,238 (42)
Female	3,636 (59)	1,683 (58)
Age at KA median, years (IQR)	55.1 (51.5–57.8)	54.9 (50.1–57.7)
Age group, years		
< 45	273 (4.5)	214 (7.3)
45–49	804 (13)	402 (14)
50–54	1,947 (32)	871 (30)
55–59	3,104 (51)	1,434 (49)
Type of implant		
TKA	4,375 (71)	2,165 (74)
UKA	1,404 (23)	466 (16)
Other/unknown	349 (5.7)	290 (9.9)
Side of operation		
Right	3,191 (52)	1,516 (52)
Left	2,937 (48)	1,405 (48)
Functional status		
Unilateral impairment	801 (60)	586 (56)
Contralateral impairment	313 (24)	256 (25)
Contralateral prosthesis	121 (9.1)	127 (12)
Other functional impairment	96 (7.2)	70 (6.7)
Region		
Capital	1,880 (31)	816 (28)
Zealand	1,353 (22)	604 (21)
Southern	1,316 (22)	557 (19)
Central	1,036 (17)	479 (16)
Northern	538 (8.8)	464 (16)
Cohabitation status		
Living alone	1,277 (21)	536 (18)
Cohabiting	4,851 (79)	2,385 (82)
Annual household income		
< 33rd percentile	541 (8.9)	317 (11)
33–67th percentile	1,390 (23)	776 (27)
> 67th percentile	4,168 (68)	1,815 (62)
Educational level		
Low	1,601 (27)	798 (28)
Medium	3,294 (55)	1,548 (54)
High	1,089 (18)	519 (18)
Labor market status 6 months before index date		
Sick leave benefits	573 (9.4)	349 (12)
Self-supporting	4,497 (73)	2,081 (71)
Other employment	432 (7.0)	223 (7.6)

Unemployment benefits	369 (6.0)	160 (5.5)
Social assistance/social security	257 (4.2)	108 (3.7)
Labor market status 4 weeks before index date		
Sick leave benefits	1,045 (17)	588 (20)
Self-supporting	4,028 (66)	1,852 (63)
Other employment	423 (6.9)	233 (8.0)
Unemployment benefits	348 (5.7)	132 (4.5)
Social assistance/social security	284 (4.6)	116 (4.0)
Employment category		
Director/chief executive	1,576 (26)	583 (27)
Employer/self-employed	259 (4.2)	119 (5.6)
Skilled worker	2,375 (39)	774 (36)
Unskilled worker	578 (9.5)	181 (8.5)
Other/unknown	1324 (22)	483 (23)
Index year		
2008	↓	780 (27)
2009	↓	826 (28)
2010	↓	861 (30)
2011	493 (8.0)	353 (12)
2012	796 (13)	43 (1.5)
2013	777 (13)	15 (0.5)
2014	790 (13)	15 (0.5)
2015	806 (13)	28 (1)
2016	754 (12)	↑
2017	845 (14)	↑
2018	867 (14)	↑
Time to RTW median (IQR), days	70 (7.0–111)	75 (7.0–126)
Proportion RTW at 24 months	5,847 (96)	2,802 (96)
Proportion competing events at 24 months	78 (2.7)	74 (1.1)

BMI: body mass index. KA: knee arthroplasty, TKA: total knee arthroplasty, UKA: unicompartmental knee arthroplasty. IQR: interquartile range, 25th; 75th percentile. Arrows indicate data merged into next cell due to low numbers.

Table S3. Supplementary characteristics of included patients, total population, and per body mass index group. Values are counts (%) unless otherwise specified

Item	Overall	Normal weight	Pre-obesity	Obesity class I	Obesity class II	Obesity class III
Number of included patients	6,128	892	2,156	1,766	880	434
Labor market status 6 months before index date						
Sick leave benefits	573 (9.4)	79 (8.9)	187 (8.7)	162 (9.2)	99 (11)	46 (11)
Self-supporting	4,497 (73)	679 (76)	1,631 (76)	1,315 (75)	591 (67)	281 (65)
Other employment	432 (7.0)	71 (8.0)	132 (6.1)	118 (6.7)	76 (8.6)	35 (8.1)
Unemployment benefits	369 (6.0)	27 (3.0)	129 (6.0)	99 (5.6)	66 (7.5)	48 (11)
Social assistance/social security	257 (4.2)	36 (4.0)	77 (3.6)	72 (4.1)	48 (5.5)	24 (5.5)
Labor market status 4 weeks before index date						
Sick leave benefits	1,045 (17)	133 (15)	350 (16)	300 (17)	174 (20)	88 (20)
Self-supporting	4,028 (66)	629 (71)	1,470 (68)	1,165 (66)	517 (59)	247 (57)
Other employment	423 (6.9)	71 (8.0)	128 (5.9)	116 (6.6)	74 (8.4)	34 (7.8)
Unemployment benefits	348 (5.7)	27 (3.0)	120 (5.6)	106 (6.0)	60 (6.8)	35 (8.1)
Social assistance/social security	284 (4.6)	32 (3.6)	88 (4.1)	79 (4.5)	55 (6.2)	30 (6.9)
Employment category						
Director/chief executive	1,576 (26)	275 (31)	597 (28)	421 (24)	196 (22)	87 (20)
Employer/self-employed	259 (4.2)	53 (5.9)	102 (4.7)	58 (3.3)	31 (3.5)	15 (3.5)
Skilled worker	2,375 (39)	319 (36)	843 (39)	718 (41)	341 (39)	154 (36)
Unskilled worker	578 (9.4)	81 (9.1)	187 (8.7)	180 (10)	82 (9.3)	48 (11)
Other/unknown	1340 (22)	163 (18)	421 (20)	383 (22)	228 (26)	129 (30)
Calendar year of KA						
2008	↓	↓	↓	↓	↓	↓
2009	↓	↓	↓	↓	↓	↓
2010	↓	↓	↓	↓	↓	↓
2011	493 (8.0)	90 (9.9)	178 (8.1)	140 (7.8)	60 (6.8)	25 (5.8)
2012	796 (13)	134 (15)	288 (13)	217 (12)	111 (13)	46 (11)
2013	777 (13)	118 (13)	275 (13)	229 (13)	95 (11)	60 (14)
2014	790 (13)	97 (11)	315 (15)	218 (12)	119 (14)	41 (9)
2015	806 (13)	129 (15)	277 (13)	210 (12)	128 (15)	62 (14)

2016	754 (12)	102 (11)	264 (12)	214 (12)	106 (12)	68 (16)
2017	845 (14)	113 (13)	283 (13)	257 (15)	137 (16)	55 (13)
2018	867 (14)	109 (12)	276 (13)	281 (16)	124 (14)	77 (18)

Grouping of body mass index (BMI) according to WHO ICD-11 classification: Normal weight and underweight (BMI < 25.0), pre-obesity (BMI 25.0–29.9), obesity class I (BMI 30.0–34.9), obesity class II (BMI 35.0–39.9), and obesity class III (BMI ≥ 40). KA: knee arthroplasty. Labor market status classified according to codes present in the Database for Registration of Marginalization at 6 months and 4 weeks prior to index date. Employment category derived from Statistics Denmark IDAP1980_2007 and IDAP2008 registers. Arrows indicate data merged into next cell due to low numbers.

Table S4. Adjusted hazard ratios (aHR)[a] with 95% confidence interval (CI) for return to work according to different body mass index groups and stratified by comorbidity index, socioeconomic position, and age group. Values include number of subjects in the cohort

	n	Normal reference	n	Pre-obesity aHR (CI)	n	Obesity class I aHR (CI)	n	Obesity class II aHR (CI)	n	Obesity class III aHR (CI)
CCI										
Low	758	1	1,866	1.1 (1.0–1.2)	1,527	1.0 (0.9–1.1)	750	0.9 (0.8–1.0)	366	1.0 (0.9–1.1)
Medium	121	1	271	0.9 (0.7–1.2)	216	1.0 (0.8–1.2)	115	1.1 (0.9–1.5)	60	1.0 (0.7–1.4)
High	13	1	19	0.8 (0.3–2.0)	23	0.8 (0.4–1.6)	15	1.0 (0.4–2.5)	8	0.3 (0.1–0.8)
Socioeconomic position										
Low	84	1	173	1.0 (0.7–1.3)	135	0.9 (0.7–1.3)	96	1.0 (0.8–1.4)	46	1.1 (0.7–1.5)
Medium	302	1	799	1.1 (1.0–1.3)	685	1.1 (0.9–1.2)	366	0.9 (0.8–1.1)	199	0.9 (0.8–1.1)
High	486	1	1,147	1.1 (0.9–1.2)	890	1.0 (0.9–1.1)	396	1.0 (0.9–1.1)	168	0.9 (0.8–1.1)
Age group										
< 45	42	1	93	1.1 (0.8–1.7)	81	1.3 (0.9–2.0)	36	1.1 (0.7–1.9)	21	0.9 (0.5–1.7)
45–49	108	1	262	1.0 (0.8–1–3)	247	1.1 (0.8–1.3)	117	1.0 (0.7–1.3)	70	0.9 (0.6–1.2)
50–54	276	1	668	1.1 (1.0–1.3)	574	1.0 (0.8–1.1)	296	1.0 (0.9–1.2)	133	1.0 (0.8–1.3)
54–59	466	1	1,133	1.0 (0.9–1.2)	864	1.0 (0.9–1.1)	431	0.9 (0.8–1.1)	210	0.9 (0.8–1.1)

[a] Adjusted hazard ratios for age group, CCI, and socioeconomic position were adjusted for sex and the two other variables. Reference: normal weight group. CCI: Charlson Comorbidity Index. BMI: body mass index. Grouping of BMI according to WHO ICD-11 classification: Normal weight and underweight (BMI < 25.0), pre-obesity (BMI 25.0–29.9), obesity class I (BMI 30.0–34.9), obesity class II (BMI 35.0–39.9), and obesity class III (BMI ≥ 40).
